# RNAseq Analysis of Rodent Spaceflight Experiments Is Confounded by Sample Collection Techniques

**DOI:** 10.1016/j.isci.2020.101733

**Published:** 2020-11-25

**Authors:** San-Huei Lai Polo, Amanda M. Saravia-Butler, Valery Boyko, Marie T. Dinh, Yi-Chun Chen, Homer Fogle, Sigrid S. Reinsch, Shayoni Ray, Kaushik Chakravarty, Oana Marcu, Rick B. Chen, Sylvain V. Costes, Jonathan M. Galazka

**Affiliations:** 1 KBR, NASA Ames Research Center, Moffett Field, CA 94035, USA; 2 NASA Ames Research Center, Moffett Field, CA 94035, USA; 3 Logyx, LLC, Mountain View, CA 94043, USA; 4 The Bionetics Corporation, NASA Ames Research Center, Moffett Field, CA 94035, USA; 5 NGM Biopharmaceuticals, South San Francisco, CA 94080, USA; 6 VeriSIM Life, San Francisco, CA 94104, USA; 7 Carl Sagan Center, SETI Institute, Mountain View, CA 94043, USA

**Keywords:** Transcriptomics, Space Sciences

## Abstract

To understand the physiological changes that occur in response to spaceflight, mice are transported to the International Space Station (ISS) and housed for variable periods of time before euthanasia on-orbit or return to Earth. Sample collection under such difficult conditions introduces confounding factors that need to be identified and addressed. We found large changes in the transcriptome of mouse tissues dissected and preserved on-orbit compared with tissues from mice euthanized on-orbit, preserved, and dissected after return to Earth. Changes due to preservation method eclipsed those between flight and ground samples, making it difficult to identify spaceflight-specific changes. Follow-on experiments to interrogate the roles of euthanasia methods, tissue and carcass preservation protocols, and library preparation methods suggested that differences due to preservation protocols are exacerbated when coupled with polyA selection. This has important implications for the interpretation of existing datasets and the design of future experiments.

## Highlights

• Experimentation is necessary to understand how organisms respond to space
• Specialized protocols are used for preserving biological samples on the ISS
• RNAseq datasets are impacted by preservation protocols used on the ISS
• Impacts can be alleviated with improved carcass preservation protocols

## Graphical Abstract

**Figure:**
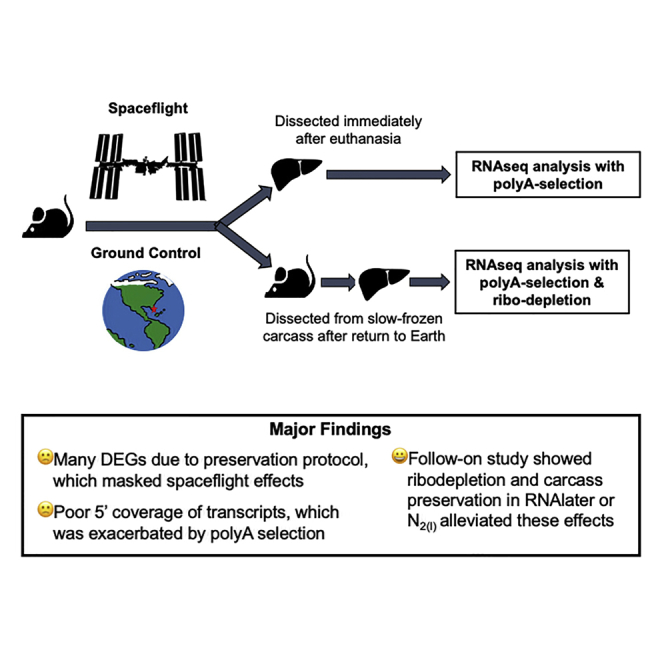

## Introduction

Spaceflight places multiple stresses upon the human body including altered gravity fields and exposure to cosmic radiation, which lead to health risks for spacefaring humans (Institute of Medicine, 2008). Decades of research on astronauts has begun to reveal how humans respond to the spaceflight environment (Garrett-Bakelman et al., 2019) but physiological monitoring of astronauts is still limited. Thus, rodent models have been essential for advancing our understanding of how mammals—including humans—respond to spaceflight. This includes the impact of spaceflight on muscle structure (Shen et al., 2017; Spatz et al., 2017; Tascher et al., 2017), liver (Beheshti et al., 2019; Jonscher et al., 2016), and immune functions (Pecaut et al., 2017; Rettig et al., 2017; Ward et al., 2018).

Despite success of the rodent model, sample collection under such difficult conditions introduces confounding factors that need to be identified and addressed. These are related to hardware limitations, small sample size, and severe restraints on astronaut crew availability. Successful experiments must work within these constraints to produce meaningful insights. In response, the first Rodent Research (RR) mission established new capabilities for conducting reliable long-duration experiments using rodents with on-orbit sample collection. Animals can either be euthanized onboard the ISS or returned to Earth alive. Both approaches introduce confounding factors. The former is experimentally challenging but preserves the sample during exposure to microgravity, whereas the latter exposes the animal to re-entry stresses, and sampling occurs only after a variable lag between landing and euthanasia—essentially sampling re-adaptation to Earth conditions in addition to the response to spaceflight. Inconsistent handling of samples necessitates a clear understanding of how dissection and preservation protocols affect downstream data generation.

We previously showed, using transcriptomic, proteomic, and immunohistochemical data from the Rodent Research-1 (RR-1), Rodent Research-3 (RR-3), and Space Transportation System (STS)-135 missions, that lipotoxic pathways are activated in rodent liver in two different strains of mice that were flown for as long as 42 days in space (Beheshti et al., 2019). Because animals in the RR-1 and RR-3 experiments were euthanized in space, this work suggested that space alone was the most likely cause for similar changes previously observed in liver samples from mice flown during the STS-135 experiments where animals returned alive to Earth (Jonscher et al., 2016). The lipotoxic effect is stronger with duration and may have ramifications for astronauts' health during long missions. This analysis did not include two flight and two ground animals from RR-1, as these animals were dissected immediately after euthanasia on-orbit as opposed to the rest, which were first returned to Earth as intact frozen carcasses for later dissection.

Here we now compare RNA-sequencing (RNAseq) datasets generated from livers preserved using these distinct protocols (Table 1). We find large changes in the transcriptome of tissues dissected and preserved on-orbit compared with tissues from mice euthanized on-orbit, preserved intact by freezing on-orbit, and dissected after return to Earth. To identify and mediate how the preservation method could have such a large effect on differential gene expression (DGE) results, we performed follow-on experiments to interrogate the role of euthanasia methods, tissue and carcass preservation protocols, and library preparation methods on DGE changes. Our findings have important implications for interpreting existing datasets and the design of future experiments.

**Table 1:** GLDS (GeneLab Data Systems) Datasets Used in This Study

GLDS #	Tissue	Study Type	Preservation Method	Library Preparation	How the Dataset was Used in the Present Study	Relevant Figures & Tables
GLDS-47	Liver	Spaceflight	Immediate	PolyA	To compare global gene expression in immediate versus carcass samples	Figure S1
GLDS-48	Liver	Spaceflight	Immediate and Carcass	PolyA	To evaluate gene expression and RNA integrity differences in immediate versus carcass samples and in samples prepared via polyA-selection versus ribo-depletion	Figures 1, 2, 3, 4, S1, S2, and S3
GLDS-49	Liver	Ground	Immediate	PolyA	To compare global gene expression in immediate versus carcass samples	Figure S1
GLDS-168	Liver	Spaceflight	Carcass	Ribodepletion	To evaluate gene expression and RNA integrity differences in samples prepared via polyA-selection vs. ribo-depletion	Figure 4
GLDS-235	Liver	Ground	Immediate and Carcass	Ribodepletion	To determine a means of carcass preservation that is most consistent with standard laboratory practices in the context of gene expression and RNA integrity and to evaluate the effects of euthanasia and tissue storage methods on gene expression	Figures 5A, 5B, S5A, S5C, S5E, and S5F, Tables 2, S3, and S4
GLDS-236	Quadriceps	Ground	Immediate and Carcass	Ribodepletion	To determine if the means of carcass preservation that is most consistent with standard laboratory practices in the context of gene expression and RNA integrity and if the effects of euthanasia on gene expression are tissue specific	Figures 5C, 5D, S5B, and S5D, Tables 3, S5, and S6

Immediate = tissues that were dissected immediately after euthanasia and Carcass = tissues that were dissected from frozen carcasses after partial thawing.

## Results

### Preservation Method Is the Primary Driver of Gene Expression Variance in RR-1 Liver Samples

**Figure 1:**
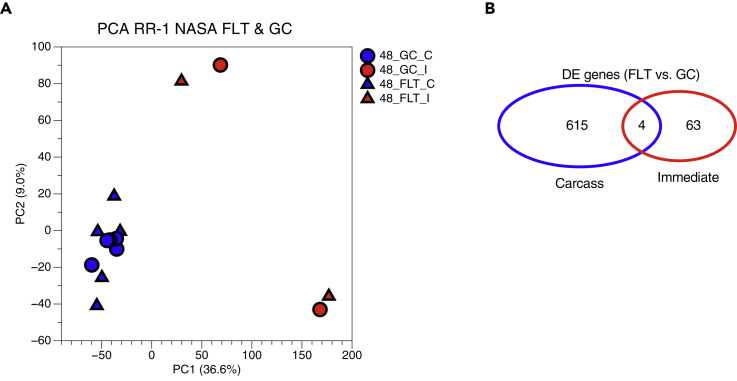
Gene Expression Differences in RR-1 NASA Spaceflight and Ground Control Liver Samples Prepared via polyA Selection (A) Principal component analysis of global gene expression in RR-1 NASA spaceflight (FLT) and respective ground control (GC) liver samples dissected immediately after euthanasia (I) or from frozen carcasses (C). Percent variance for each principal component (PC) is shown. (B) Venn diagram showing the number of similar and unique differentially expressed genes, spaceflight (FLT) versus ground control (GC), in Carcass (blue) and Immediate (red) samples (adj. p value < 0.05). Data are from GLDS-48.

**Figure 2:**
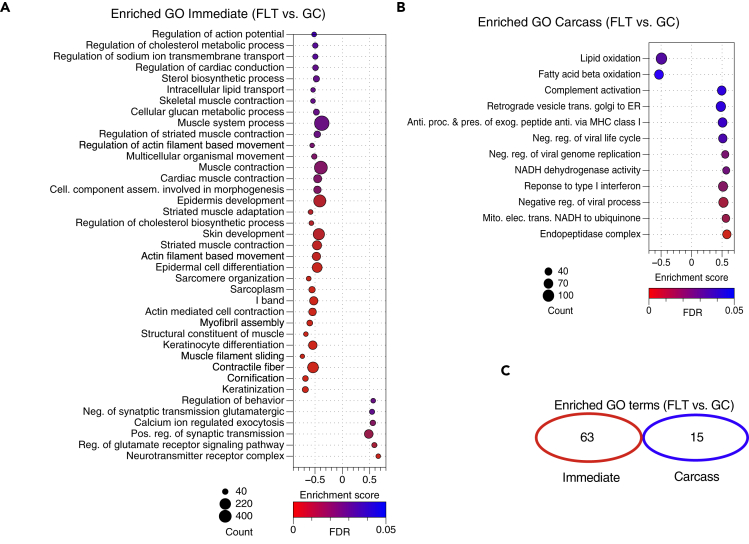
Enriched Gene Ontology (GO) Terms between the RR-1 NASA Spaceflight and Ground Control Groups (A) Enriched GO terms between the spaceflight (FLT) and ground control (GC) immediate samples identified by Gene Set Enrichment Analysis (gene set permutation). (B) Enriched GO terms between the spaceflight (FLT) and ground control (GC) carcass samples identified by Gene Set Enrichment Analysis (gene set permutation). In both A and B, the more positive or negative the enrichment scores, the higher the expression in spaceflight or ground control samples, respectively. Dot size indicates number of genes within each GO term. Dot color indicates false discovery rate (FDR). GO terms displayed met the thresholds of FDR <0.05, NOM p < 0.01, gene set size >40. (C) Venn diagram of the number of enriched GO terms identified in Carcass (blue) and Immediate (red) samples when comparing spaceflight (FLT) and ground control (GC) samples. GO terms in Venn diagram met the threshold of FDR <0.05, NOM p < 0.01. Data are from GLDS-48.

To assess gene expression differences in liver samples from the RR-1 NASA Validation mission (Cadena et al., 2019; Globus and Galazka, 2015; Ronca et al., 2019), RNA was extracted from livers dissected from spaceflight (FLT) and ground control (GC) animals either immediately after euthanasia (immediate preservation, I) or from frozen carcasses after partial thawing (carcass preservation, C) and sequenced following polyA selection (Figure S1). Principal component analysis (PCA) revealed preservation method (C versus I) as the primary driver of variance among samples rather than spaceflight (FLT versus GC) (Figure 1A). Furthermore, there was an order of magnitude difference in the number of differentially expressed genes (DEGs) identified in FLT versus GC carcass samples than was observed in FLT versus GC immediate samples, and only 4 DEGs overlapped between the two preservation methods (Figure 1B). Gene set enrichment analysis of FLT versus GC immediate- (Figure 2A) and carcass-derived (Figure 2B) samples showed no overlap in enriched gene ontology (GO) terms (Figure 2C), showing that any gene expression changes in the liver as a result of spaceflight exposure were confounded by the sample preservation method used.

Since livers from only 2 FLT and 2 GC animals in the RR-1 NASA Validation mission were preserved via the immediate method, RNA from livers prepared via the immediate method from two additional studies, the RR-1 CASIS mission (Figure S1) (Cadena et al., 2019; Globus et al., 2015; Ronca et al., 2019) and a ground-based preservation and storage study (Figure S2) (Choi et al., 2016; GeneLab, 2016), were also sequenced following polyA selection. Despite multiple different experimental factors in RR-1 NASA, RR-1 CASIS, and the ground-based preservation studies, PCA continued to show preservation method as the primary driver of variance among samples in these datasets (Figure S3).

### Carcass-Preserved Samples Exhibit Less Uniform Transcript Coverage than Immediate-Preserved Samples

**Figure 3:**
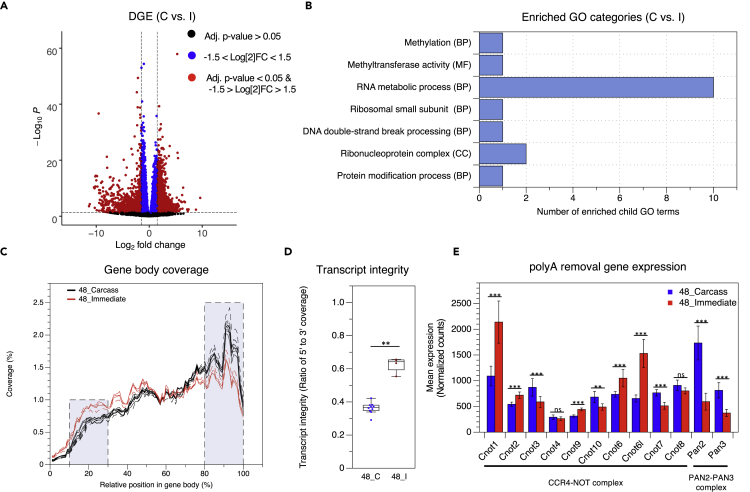
Gene Expression Changes and Transcript Integrity in Carcass versus Immediate RR-1 NASA Liver Samples (A) Volcano plot showing 2,934 differentially expressed genes in all carcass (both spaceflight and ground control) versus all immediate (both spaceflight and ground control) samples (adj. p value < 0.05 and 1.5 < Log2 fold change < −1.5). (B) Common parent terms of enriched gene ontology (GO) terms identified by Gene Set Enrichment Analysis of all carcass (C) versus all immediate (I) samples (phenotype normalized, FDR <0.3, NOM p < 0.01). (C) Gene body coverage in Carcass (black) and Immediate (red) spaceflight and ground control samples. (D) The percent coverage of the 5′- and 3′-shaded regions in panel C were used to calculate the 5′ to 3′ transcript integrity ratio for each sample. All Carcass (blue) and Immediate (red) samples are grouped together (∗∗ = p < 0.01, Mann–Whitney U test). (E) Average expression of polyA removal genes in Carcass (blue) and Immediate (red) groups from RNAseq data. Cnot1, Cnot2, Cnot3, Cnot4, Cnot9, Cnot10, Cnot6, Cnot6l, Cnot7, and Cnot8 are part of the CCR4-NOT complex. Pan2 and Pan3 are part of the PAN2-PAN3 complex. Error bars indicate standard deviation (∗ = adj. p < 0.05, ∗∗ = adj. p < 0.01, ∗∗∗ = adj. p < 0.001, ns = not significant, Wald test). Data are from GLDS-48.

To further investigate the observed differences in preservation method, RR-1 NASA FLT and GC liver samples derived from the carcass preservation method were grouped together (C) and FLT and GC liver samples derived from the immediate preservation method were grouped together (I). DGE was evaluated in carcass versus immediate samples. Many more genes were differentially expressed in carcass versus immediate samples (2,934 DEGs) than in either FLT-C versus GC-C (619 DEGs) or in FLT-I versus GC-I (67 DEGs) samples, further supporting preservation method as the primary driver of variance in RR-1 NASA liver samples (Figure 3A). Gene set enrichment analysis revealed that several of the gene ontologies enriched in carcass samples (when compared with immediate samples) involved RNA regulation and processing (Figure 3B). Despite similarly high RNA Integrity Number (RIN) values (Figure S4), carcass samples exhibited significantly less 5′ to 3′ gene body coverage than immediate samples as indicated by their 5′ to 3′ transcript integrity ratios. The 5′ to 3′ transcript integrity ratios were determined by dividing the percent coverage indicated in the 5′-shaded region by that in the 3′-shaded region for each sample (Figures 3C and D).

### Expression of Genes Involved in 5′-Methylguanosine Decapping and PolyA Removal Is Affected by Preservation Condition

Given the differences in gene body coverage between carcass and immediate samples, we evaluated the expression of 5′-methylguanosine decapping and polyA removal genes in these groups from the RNAseq data. In mammals, eight genes have decapping activity *in vitro* and/or in cells: Dcp2 (Nudt20), Nudt3, Nudt16, Nudt2, Nudt12, Nudt15, Nudt17, Nudt19. In addition, Dxo acts on partially capped mRNAs (Grudzien-Nogalska and Kiledjian, 2017). Two of these genes—Dxo and Nudt2—were significantly more expressed in the carcass samples, whereas another two—Nudt15 and Dcp2 (Nudt20)—were significantly more expressed in immediate samples (Figure S5). Removal of polyA tails from mRNA is catalyzed by two complexes. The first, CCR4-NOT, consists of CNOT1, CNOT2, CNOT3, CNOT4, CNOT9, CNOT10, CNOT6, CNOT6L, CNOT7, and CNOT8. The second, PAN2-PAN3, consists of PAN2 and PAN3 (Siwaszek et al., 2014). In the case of the 10 subunit CCR4-NOT complex, we observed 5 genes that were more highly expressed in the immediate group (Cnot1, Cnot2, Cnot9, Cnot6, Cnot6L) and 3 that were more highly expressed in the carcass group (Cnot3, Cnot10, Cnot7) (Figure 3E). In the case of the PAN2-PAN3 complex, both Pan2 and Pan3 were more highly expressed in the carcass group (Figure 3E).

### Samples Sequenced Following Ribodepletion Exhibit More Uniform Transcript Coverage than Samples Prepared with PolyA Selection

**Figure 4:**
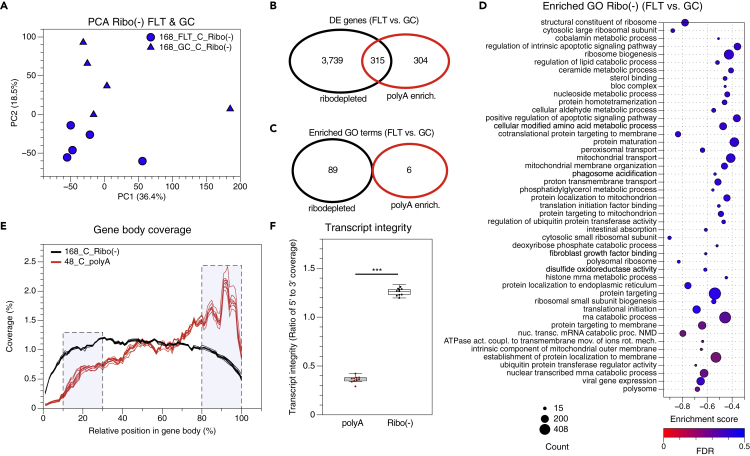
Evaluation of Gene Expression and Transcript Integrity in RR-1 NASA Carcass-dissected FLT and GC Samples Prepared via polyA Selection and Ribodepletion Methods (A) Principal component analysis of global gene expression in RR-1 NASA spaceflight (FLT) and ground control (GC) liver samples dissected from frozen carcasses (C) and prepared via ribodepletion (Ribo(−)). Percent variance for each principal component (PC) are shown. (B) Venn diagram of differentially expressed (DE) genes between spaceflight (FLT) and ground control (GC) samples prepared with ribodepletion (black) or polyA selection (red) methods (adj. p value < 0.05). (C) Venn diagram of the number of similar and unique enriched gene ontology (GO) terms (spaceflight (FLT) versus ground control (GC)) identified in ribodepleted (black) and polyA selected (red) prepared samples (NOM p < 0.01, FDR <0.5, phenotype permutation). (D) Enriched GO terms between the spaceflight (FLT) and ground control (GC) carcass samples prepared with ribodepletion identified by Gene Set Enrichment Analysis (phenotype permutation). The more positive or negative the enrichment scores, the higher the expression in spaceflight or ground control samples, respectively. Dot size indicates number of genes within GO term. Dot color indicates false discovery rate (FDR). GO terms displayed met the thresholds of FDR <0.5, NOM p < 0.01, 1.6 < NES < −1.6. (E) Gene body coverage of ribodepleted (Ribo(−)) and polyA selected (polyA) spaceflight and ground control carcass (C) samples. (F) The percent coverage of the 5′- and 3′-shaded regions in panel E were used to calculate the transcript integrity ratio for each sample (∗∗∗ = p < 0.001, Mann–Whitney U test). Data are from GLDS-48 and -168.

The polyA selection library preparation method, which was initially used to evaluate gene expression differences in RR-1 NASA Validation mission liver samples, requires intact RNA to minimize bias (Kumar et al., 2017; Petrova et al., 2017). Because our data suggest that the carcass samples were more degraded than the immediate samples (Figures 3C and 3D), the total RNA isolated from the RR-1 NASA Validation mission carcass liver samples was used to prepare libraries with the ribodepletion method to minimize transcript integrity bias, then re-sequenced. PCA showed a more distinct separation of FLT and GC carcass samples when the samples were prepared via the ribodepletion method (Figure 4A) than by polyA selection (FLT-C and GC-C samples in Figure 1A). DEGs were identified in FLT versus GC carcass samples prepared with the ribodepletion method and compared with those from polyA-prepared carcass samples. Although hundreds of DEGs in FLT versus GC carcass samples overlap between ribodepleted and polyA-prepared samples, more DEGs were identified in FLT versus GC samples prepared with the ribodepletion method (Figure 4B), suggesting this method may be more sensitive. There was no overlap of enriched gene ontology terms in FLT versus GC samples processed by ribodepletion and polyA enrichment (Figures 4C and 4D).

Next, transcript integrity was evaluated in the ribodepletion-prepared FLT and GC carcass samples and compared with polyA-selection-prepared carcass samples. Samples prepared with the polyA selection method exhibited less coverage of the 5′ portion of transcripts compared with ribodepletion-prepared samples (Figures 4E and 4F). Thus, ribodepletion was used to further investigate the effects of preservation method (Carcass versus Immediate) on gene-expression in a ground-based tissue preservation study.

### Total RNA Sequencing Mitigates the Impact of Preservation Method on Gene Expression Changes in the Liver

We designed a ground-based tissue preservation study to determine the best approach to mitigate the impact of preservation method on gene expression and to identify other confounding variables important for interpreting data from other RR missions. We tested euthanasia and preservation techniques used in different RR missions and compared them with standard laboratory protocols for tissue preservation. In addition to liver samples, we also analyzed quadriceps to determine whether sample preservation methods also confounded DGE analysis in this tissue.

Mice of the same age, sex, strain, and source as those used in the RR-1 NASA Validation mission were used in the ground-based tissue preservation study. Mice were evenly divided into one of six groups as shown in Figure S6. The mice in groups 1–4 were euthanized with pentobarbital/phenytoin (Euthasol) as in RR-1 (Choi et al., 2020), then subjected to various preservation protocols to evaluate the phenomena observed in RR-1 NASA carcass and immediate liver samples. Livers and quadriceps were dissected immediately after euthanasia from mice in group 1. These tissues were divided into thirds and preserved in one of three ways: (1) freezing in dry ice to mimic the cold stowage container that was used to freeze the immediate liver samples in the RR-1 mission, (2) submersion in LN2, or (3) with RNA*later*. After preservation, all tissues were stored at −80°C until further processing.

Although it is a common practice to dissect mice immediately after euthanasia, due to limitations in crew time for spaceflight experiments, immediate dissection is not always possible. Thus, most tissues are preserved *in situ* within the carcass. We therefore sought to determine the most effective way to preserve carcasses that would minimize unintended gene expression changes in tissues preserved *in situ*. Mice in groups 2–4 were used to test three different carcass preservation methods: (1) slow freezing in dry ice (DI) to mimic the most common method of carcass preservation used in RR missions to date, (2) snap freezing by submersion in liquid nitrogen (LN2), and (3) segmenting the carcass into thirds and preserving in RNA*later*, mimicking the preservation method used in the Rodent Research-7 mission. After preservation, all carcasses were stored at −80°C until further processing.

Carcasses from mice in groups 2–4 were partially thawed, and quadriceps and livers were dissected, then snap frozen, and stored at −80°C until RNA extraction to mimic the protocol most commonly implemented when carcasses return from spaceflight missions, including RR-1. A summary of all liver and quadriceps tissues evaluated in the present ground-based tissue preservation study are summarized in Tables S1 and S2, respectively. Total RNA was extracted from all liver and quadriceps tissue samples and prepared for sequencing using the ribodepletion method and sequenced.

**Figure 5:**
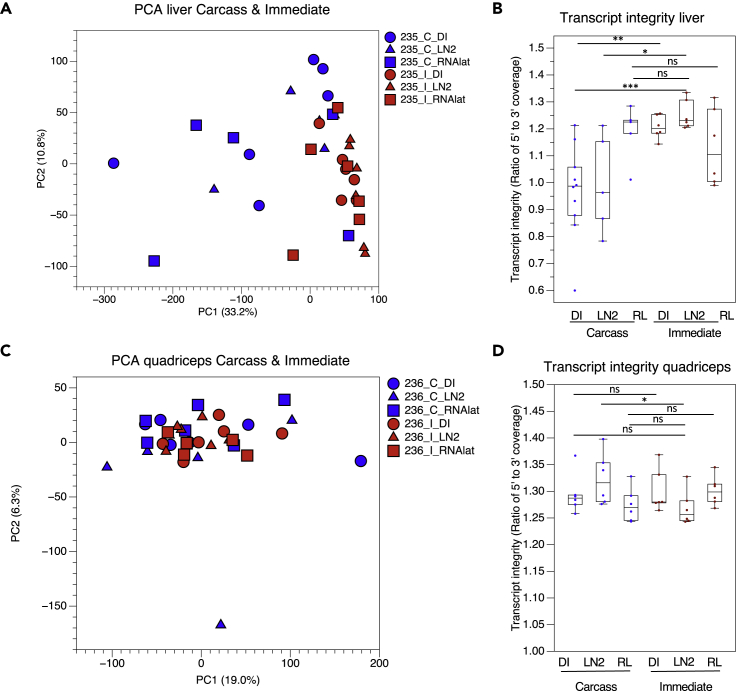
Gene Expression and Transcript Integrity Analysis of Preservation Methods for Liver and Quadriceps Samples Liver and quadriceps samples were dissected from mice immediately after euthanasia with pentobarbital/phenytoin (Euthasol) then preserved in dry ice (I_DI), liquid nitrogen (I_LN2), or RNA*later* (I_RNAlat) before RNAseq analysis. Alternatively, liver and quadriceps samples were dissected from partially thawed frozen carcasses of mice that were euthanized with pentobarbital/phenytoin (Euthasol) and then preserved in dry ice (C_DI), liquid nitrogen (C_LN2), or segmented into thirds and preserved in RNA*later* (C_RNAlat) before RNAseq analysis. (A) Principal component analysis of liver samples. Percent variance for each principal component (PC) is shown. (B) Uniformity of gene body coverage in liver samples. (C) Principal component analysis of quadriceps samples. Percent variance for each principal component (PC) is shown. (D) Uniformity of gene body coverage in quadriceps samples. (∗∗∗ = p < 0.001, ∗∗ = p < 0.01, ∗ = p < 0.05, ns = no significance, Mann–Whitney U test). Data are from GLDS-235 (liver) and -236 (quadriceps).

Global gene expression and transcript integrity were evaluated in liver samples from groups 1–4 to identify differences in DGE resulting from the carcass and immediate preservation protocols. PCA showed overlap among immediate samples despite differences in tissue preservation methods (Figure 5A). Similar to RR-1 carcass and immediate samples (Figures 1A and S3), the dry ice-preserved carcass and immediate samples, which mimic the RR-1 preservation conditions, clustered away from each other, albeit to a much lesser degree than that observed with the polyA-prepared RR-1 samples (Figure 5A). Furthermore, the dry-ice-preserved carcass samples exhibited less 5′ gene body coverage than the dry-ice-preserved immediate samples (Figure 5B). Although this observation is consistent with that observed in RR-1 carcass and immediate samples (prepared using the polyA selection method) (Figures 3C and 3D), the difference was less dramatic. Therefore, using the ribodepletion method appears to partially alleviate the differences observed in transcript integrity between carcass and immediate samples.

### Carcass Preservation by LN2 or RNAlater Immersion Most Closely Mimic Standard Tissue Preservation Protocols

Livers dissected from carcasses preserved in either RNA*later* or LN2 exhibit more overlap with immediate preserved liver samples than those from carcasses preserved in dry ice (Figure 5A). Unlike livers dissected from slow (dry ice) or snap (LN2) frozen carcasses, livers dissected from carcasses preserved in RNA*later* showed no difference in 5′ to 3′ transcript coverage when compared with livers dissected immediately after euthanasia (Figure 5B). These data suggest that carcass segmentation and preservation in RNA*later* may protect the liver from transcript degradation when preserved *in situ*.

We next assessed the effects of various carcass freezing methods on gene expression changes in the liver when compared with livers that were dissected immediately after euthanasia, then preserved in either RNA*later* or LN2. Only a few genes were differentially expressed between livers dissected immediately and preserved on dry ice, in RNA*later*, or in LN2 (Table S3). Similarly, pairwise gene set enrichment analysis showed no significantly enriched GO terms between these tissue preservation methods (Table S3), suggesting that for immediately dissected livers, the tissue preservation method had minimal impact on gene expression. Livers dissected from slow (dry ice) frozen carcasses, which most closely mimics the carcass preservation method used in RR-1 NASA Validation and several other RR missions (including RR-3 and Rodent Research-6), exhibited the most DEGs when compared with immediately dissected livers preserved in either LN2 or RNA*later* (Tables 2 and S4, respectively). In contrast, livers dissected from carcasses preserved in either RNA*later* or LN2 exhibited hundreds fewer DEGs when compared with immediately dissected livers preserved in either LN2 or RNA*later* (Tables 2 and S4, respectively). These data indicate that carcass segmentation and preservation in RNA*later* or preserving carcasses by submersion in LN2 more closely mimic the common preservation methods used in terrestrial laboratories, than does the slow freeze carcass preservation method used in the RR-1 NASA Validation study.

**Table 2:** Comparisons of Carcass Preservation Methods to Immediate Liquid Nitrogen Method on Gene Expression in Livers

Comparison	# DEG (adj. p < 0.05)	# DEG (adj. p < 0.05 & |Log2 FC| > 1.5)	# Enriched GO Terms (NOM p < 0.01)	# Enriched GO Terms (FDR <0.5 & NOM p < 0.01)	# Enriched GO Terms (FDR <0.25 & NOM p < 0.01)
Euth_C_DI (n = 6) vs. Euth_I_LN2 (n = 6)	3798	3143	16, 0	0, 0	0, 0
Euth_C_LN2 (n = 5) vs. Euth_I_LN2 (n = 6)	784	515	30, 0	0, 0	0, 0
Euth_C_RL (n = 5) vs. Euth_I_LN2 (n = 6)	2118	1952	22, 0	0, 0	0, 0

The number of differentially expressed genes (DEG) and enriched gene ontology (GO) terms identified by Gene Set Enrichment Analysis (phenotype permutation) were evaluated pairwise in liver samples from different carcass preservation methods compared with immediate samples preserved in liquid nitrogen. For GO terms, number on the left corresponds to the group to the left of the “versus”, and number on the right corresponds to the group to the right of the “versus” in the “Comparison” column. n numbers, p values, log2 fold changes, and FDR values are indicated. Euth = euthanasia by pentobarbital/phenytoin, I = tissue dissected immediately after euthanasia, C = tissue dissected from frozen carcass that has been partially thawed, DI = dry ice, LN2 = liquid nitrogen, RL = RNA*later*. Data are from GLDS-235.

### The Impact of Preservation Method on Gene Expression Is Tissue Dependent

To determine if the observed differences in gene expression due to carcass preservation method are unique to the liver, gene expression and transcript integrity were also evaluated in quadriceps from mice in groups 1–4 (Figure S6 and Table S2). PCA showed more overlap among carcass and immediate quadriceps samples (Figure 5C) than among carcass and immediate liver samples (Figure 5A), suggesting that gene expression in the quadriceps is less sensitive to preservation methods. Unlike liver samples, almost no significant differences were observed in 5′ to 3′ gene body coverage in quadriceps samples prepared using different preservation methods (Figure 5D).

Fewer DEGs were identified in carcass versus immediate quadriceps samples than carcass versus immediate liver samples for almost every preservation method tested (Tables 3, S5, and S6), further supporting that gene expression in the quadriceps is less sensitive to different types of preservation methods. Although there are fewer differences over-all, similar to what was observed in liver samples, cutting the carcass into thirds and then preserving in RNA*later* resulted in the fewest DEGs when compared with immediate dissection followed by tissue preservation in LN2 or RNA*later* (Tables 3 and S6, respectively).

**Table 3:** Comparisons of Carcass Preservation Methods to Immediate Liquid Nitrogen Method on Gene Expression in Quadriceps

Comparison	# DEG (adj. p < 0.05)	# DEG (adj. p < 0.05 & |Log2 FC| > 1.5)	# Enriched GO Terms (NOM p < 0.01)	# Enriched GO Terms (FDR <0.5 & NOM p < 0.01)	# Enriched GO Terms (FDR <0.25 & NOM p < 0.01)
Euth_C_DI (n = 6) vs. Euth_I_LN2 (n = 6)	2	0	15, 17	0, 0	0, 0
Euth_C_LN2 (n = 6) vs. Euth_I_LN2 (n = 6)	36	24	24, 24	0, 22	0, 11 (26 with p < 0.05)
Euth_C_RL (n = 6) vs. Euth_I_LN2 (n = 6)	2	2	30, 9	1, 2	0, 0

The number of differentially expressed genes (DEG) and enriched gene ontology (GO) terms identified by Gene Set Enrichment Analysis (phenotype permutation) were evaluated pairwise in quadriceps samples from different carcass preservation methods compared with immediate samples preserved in liquid nitrogen. For GO terms, the first number corresponds to the group to the left of the “versus”, and the second number corresponds to the group to the right of the “versus” in the “Comparison” column. n numbers, p values, log2 fold changes, and FDR values are indicated. Euth = euthanasia by pentobarbital/phenytoin, I = tissue dissected immediately after euthanasia, C = tissue dissected from frozen carcass that has been partially thawed, DI = dry ice, LN2 = liquid nitrogen, RL = RNA*later*. Data are from GLDS-236.

### Gene Expression in Select Tissues Was Not Affected by the Method of Euthanasia

Because the most common euthanasia method used in RR missions to date is intraperitoneal (IP) injection of ketamine/xylazine and the most common euthanasia method used in standard laboratories is CO_2_ inhalation, these methods were used to euthanize mice in groups 5 and 6, respectively, to determine if euthanasia method is another confounding variable that could affect gene expression in select tissues (Figure S6, Tables S1 and S2). Gene expression was evaluated in livers and quadriceps dissected from mice in groups 2, 5, and 6 (Figures S7A–S7D). PCA showed no distinct differences in global gene expression in liver (Figure S7A) or quadriceps (Figure S7B) samples dissected from mice euthanized with different methods. Pairwise differential gene expression analysis and gene set enrichment analysis also identified few, if any, DEGs and enriched GO terms among liver (Figure S7C) and quadriceps (Figure S7D) samples. These data suggest that the types of euthanasia methods evaluated here do not impact gene expression in select tissues.

## Discussion

Herein, we show that protocols used to preserve mouse carcasses on-orbit have large effects on gene expression patterns as measured by RNAseq. Indeed, changes in gene expression due to preservation condition overwhelmed those due to spaceflight. Gene set enrichment analysis showed that many GO terms enriched due to carcass preservation were involved in RNA processing. This correlated with reduced transcript integrity (relatively poor coverage of the 5′ end of transcripts) in samples from carcasses preserved on-orbit when these were sequenced with a polyA enrichment RNAseq protocol.

Although RNAseq following polyA selection can more efficiently quantify gene expression (Kumar et al., 2017), ribodepletion methods are more effective on degraded RNA samples (Li et al., 2014; Schuierer et al., 2017). However, although the RNA used in this study was of good quality (RIN >7), we observed a severe bias in transcript coverage following polyA selection depending upon the tissue preservation condition utilized. Specifically, samples taken from carcasses that were slow-frozen on-orbit exhibited a lower 5′ to 3′ coverage ratio as compared with samples taken from immediately dissected tissues. Although resequencing of the carcass flight samples with a ribodepletion protocol produced a more even 5′ to 3′ coverage ratio, our follow-on studies that directly compared slow carcass freezing with immediate dissection revealed a similar (albeit reduced) 5′ to 3′ coverage bias. Taken together, this suggests that slow carcass freezing causes transcript degradation that in-turn leads to reduced 5′ coverage.

mRNA degradation starts with the removal of the polyA tail, at which point degradation continues either from the 3′ end *via* the exosome complex or the 5′ end following removal of the 5′-methylguanosine cap. Deadenylation of cytoplasmic mRNA is the rate-limiting step in mRNA degradation and is catalyzed by one of two complexes: the CCR4-NOT complex, which consists of 10 subunits (CNOT1, CNOT2, CNOT3, CNOT4, CNOT6, CNOT6l, CNOT7, CNOT8, CNOT9, CNOT10), and the PAN2-PAN3 deadenylation complex consisting of two subunits (PAN2, PAN3) (Siwaszek et al., 2014). We observed transcriptional changes to multiple subunits in each of these complexes when comparing carcass and immediate samples. Most striking was the coordinated upregulation of both Pan2 and Pan3 in the carcass samples from the RR-1 NASA Validation mission, which suggests an increase in PAN2-PAN3 deadenylation activity, which could result in loss of polyA tails in some transcripts. This could lead to poor mRNA capture by our polyA enrichment protocol and result in some of the differences seen between the polyA enrichment and ribodepletion protocols.

Three proteins—Dcp2 (Nudt20), Nudt3, Nudt16—have decapping activity both *in vitro* and in cells, whereas an additional five—Nudt2, Nudt12, Nudt15, Nudt17, Nudt19—have decapping activity *in vitro*. In addition, the Dxo family of proteins acts on partially capped mRNAs (Grudzien-Nogalska and Kiledjian, 2017). Although regulation of these proteins is complex and involves subcellular localization and post-translation modification, we observed evidence for altered expression of these decapping enzymes: Dxo and Nudt2 were more abundant in carcass samples, whereas Nudt15 and Dcp2 (Nudt20) were more abundant in immediate samples. Although these changes are not coordinated, they do point to altered decapping activity within the carcass samples. As decapping precedes mRNA degradation via the 5′ exonuclease, XRN1, this could alternatively explain the relatively poor 5′ transcript coverage seen in both polyA enriched and ribodepleted carcass liver samples. Additional experimentation will be necessary to confirm the changes to decapping and deadenylation enzymes seen here and to understand their role in the 5′ to 3′ coverage bias observed.

We observed a marked difference in the 5′ to 3′ coverage bias between liver and quadriceps samples. Although liver samples were sensitive to carcass preservation via slow- or snap-freeze, quadriceps samples were not. There are a number of possible explanations for this. First, it could be due to the surface exposure of the quadriceps, which would lead to more rapid quenching of biochemical processes. Second, inherent differences in the transcript pool, mRNA half-lives, and enzymatic complement of liver and quadriceps could offer a biological answer. Although we cannot distinguish between these mechanisms, our observations are consistent with previous results showing that postmortem changes to mRNA is tissue dependent (Inoue et al., 2002; Lee et al., 2005; Miyatake et al., 2004).

The poor transcript integrity in slow-frozen carcasses sequenced with a polyA enrichment protocol was not evident in pre-sequencing QC analyses. Indeed, all samples had RIN values >7, and there was no correlation between the gene expression differences and RIN. This distinguishes our results from previous studies showing a strong correlation between RIN values and loss of 5′ coverage (Davila et al., 2016; Sigurgeirsson et al., 2014). Therefore, additional pre-sequencing QC analyses capable of detecting these issues would be useful. Low throughput sequencing is rapid, decreasing in cost, and being adopted as a QC step but does not provide the coverage necessary to detect the biases seen here.

Alternatively, if effective pre-sequencing QC metrics cannot be developed, a number of analytical approaches could be utilized. In one category are methods that calculate additional metrics such as mRIN (Feng et al., 2015) and TIN (Wang et al., 2016) to allow assessment of transcript integrity and exclusion of problematic samples. In a second category are processes that account for variable transcript integrity by considering only reads that occur near the 3′ end of transcripts (Sigurgeirsson et al., 2014), controlling for the effects of RIN using a linear model framework (Gallego Romero et al., 2014), or by calculating idealized coverage curves on a gene-by-gene basis and using these for normalization (Xiong et al., 2019). Additional analyses are necessary to determine if these approaches can mitigate the issue observed here.

**Figure 6:**
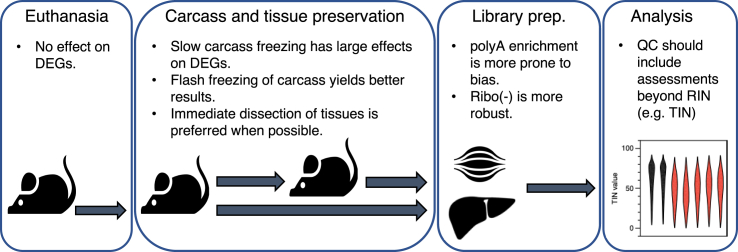
Sample Preservation and Preparation Recommendations Recommendations are provided for euthanasia, carcass and tissue preservation, library preparation, and quality control (QC) analysis methods based on observations made from liver and quadricep samples.

Although we do not have a complete picture of the mechanisms resulting in the apparent gene expression change resulting from slow carcass freezing, we were able to identify effective mitigation strategies (our suggestions are summarized in Figure 6). Foremost among these is the utilization of a ribodepletion protocol in place of polyA enrichment. In this study, ribodepletion resulted in more even gene body coverage and was not as sensitive to slow freezing of carcasses. This is in agreement with previous studies that found that ribodepletion is less prone to bias introduced by poor RNA quality (Li et al., 2014) and less prone to 3′ coverage bias (Schuierer et al., 2017). Beyond this, we found that two carcass preservation methods generated acceptable results, with few DEGs and enriched GO terms when compared with the immediate dissection of tissues and preservation in liquid nitrogen—the *de facto* gold standard. The first is rapid freeze of carcasses in liquid nitrogen and subsequent storage at −80°C, followed by partial thaw, dissection, and tissue preservation in liquid nitrogen. Although this led to some loss of 5′ transcript coverage, it had the fewest DEGs (515, adj. p < 0.05 & |Log2 FC| > 1.5) and no enriched GO terms (FDR <0.25, NOM <0.01) when compared with immediate dissection. Alternatively, segmentation of carcasses and immersion in RNA*later* and subsequent storage at −80°C, followed by partial thaw, dissection, and tissue preservation in liquid nitrogen resulted in better maintenance of 5′ transcript coverage but an increased number of DEGs (1952, adj. p < 0.05 & |Log2 FC| > 1.5), although no GO terms were enriched (FDR <0.25, NOM <0.01). As euthanasia protocols can change serum biomarkers (Pierozan et al., 2017) and mRNA expression levels (Staib-Lasarzik et al., 2014), we were reassured to find that the euthanasia protocols used here did not affect gene expression in liver or quadriceps.

To conclude, our results indicate that care must be taken in choosing sample preservation protocols that preserve transcriptional patterns and other embedded information but that are also feasible in resource-constrained environments such as those found in space.

### Limitations of the Study

This study directly assessed the effects of preservation protocols on mouse liver and quadriceps, thus extrapolation to other tissues should be done with caution. Moreover, this study is not capable of identifying the molecular mechanisms responsible for the observed effects, and additional experimentation is required to confirm that the changes to decapping enzyme expression are responsible for changes in transcript integrity and apparent gene expression.

### Resource Availability

#### Lead Contact

Jonathan M. Galazka, NASA Ames Research Center, jonathan.m.galazka@nasa.gov.

#### Materials Availability

This study did not generate unique materials. When applicable, excess RNA is available for request from the NASA Institutional Scientific Collection (https://www.nasa.gov/ames/research/space-biosciences/isc-bsp).

#### Data and Code Availability

All sequencing data are available at NASA GeneLab (www.genelab.nasa.gov) (Ray et al., 2019). polyA-enrichment-based data from the RR-1 NASA Validation mission samples are at GLDS-48 (Globus and Galazka, 2015). polyA-enrichment-based data from the RR-1 CASIS samples are at GLDS-47 (Globus et al., 2015). Data from ground-based study of tissue storage conditions are at GLDS-49 (GeneLab, 2016). Ribodepletion-based resequencing data from the RR-1 NASA Validation mission samples are at GLDS-168 (Galazka, 2018). Liver data from ground-based freezing study are at GLDS-235 (Galazka, 2019a). Quadriceps data from ground-based freezing study are at GLDS-236 (Galazka, 2019b). Raw data used to generate all PCA, DGE, GSEA, and gene body coverage analyses shown here are available on Mendeley Data: https://doi.org/10.17632/5hzrgfgxct.1. Scripts used for processing RNAseq data are available at https://github.com/nasa/GeneLab_Data_Processing.

## Methods

All methods can be found in the accompanying Transparent Methods supplemental file.

## Supplemental Information

Document S1. Transparent Methods, Figures S1–S7, Tables S1–S6
