# Supplementary material for: RNAseq Analysis of Rodent Spaceflight Experiments Is Confounded by Sample Collection Techniques
Source: iScience. 2020 Nov 25;23(12):101733. doi: 10.1016/j.isci.2020.101733 (PMC7756143; doi:10.1016/j.isci.2020.101733)
Supplement: Document S1. Transparent Methods, Figures S1–S7, Tables S1–S6 [file mmc1.pdf]

iScience, Volume *23*

Supplemental Information

RNAseq Analysis of Rodent Spaceflight Experiments Is Confounded by Sample Collection Techniques

San-Huei Lai Polo, Amanda M. Saravia-Butler, Valery Boyko, Marie T. Dinh, Yi-Chun Chen, Homer Fogle, Sigrid S. Reinsch, Shayoni Ray, Kaushik Chakravarty, Oana Marcu, Rick B. Chen, Sylvain V. Costes, and Jonathan M. Galazka

Supplemental Figures

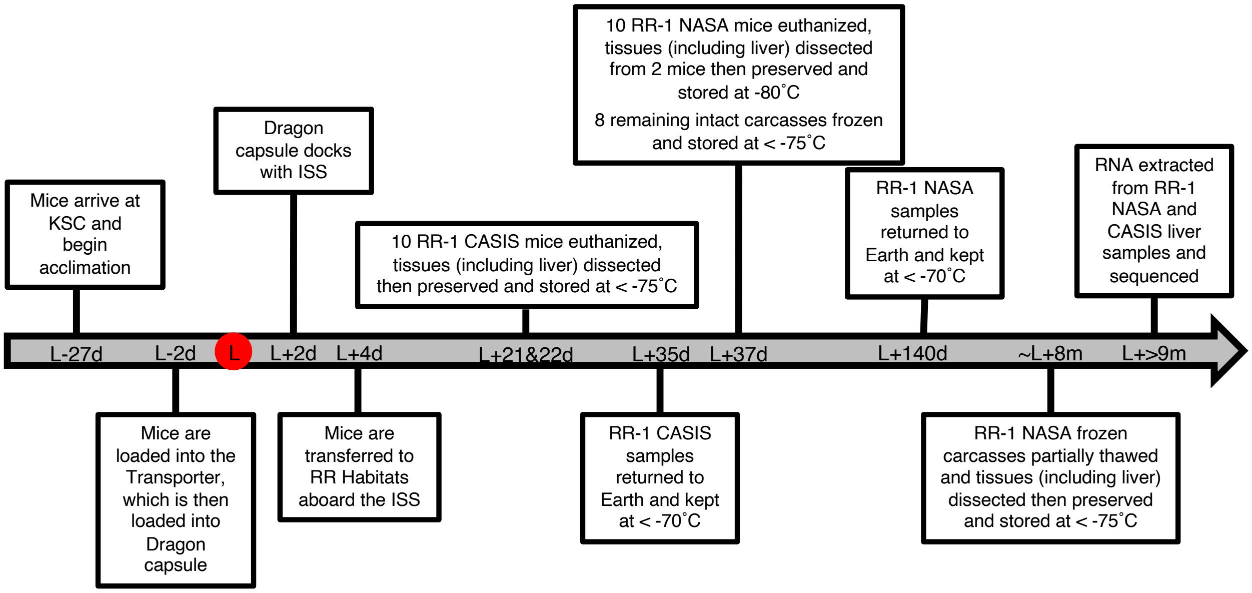

Figure S1. RR-1 mission timeline. Related to Figures 1-4. Timeline indicating major events in the Rodent Research-1 (RR-1) mission relative to the launch date (L). A minus sign (-) indicates time in days (d) before launch and a plus sign (+) indicates time in days (d) or months (m) after launch. Age-matched ground control animals were processed on similar timeline but on a 4-day delay to mimic spaceflight conditions. KSC = Kennedy Space Center; ISS = International Space Station; CASIS = Center for the Advancement of Science in Space; NASA = National Aeronautics and Space Administration.

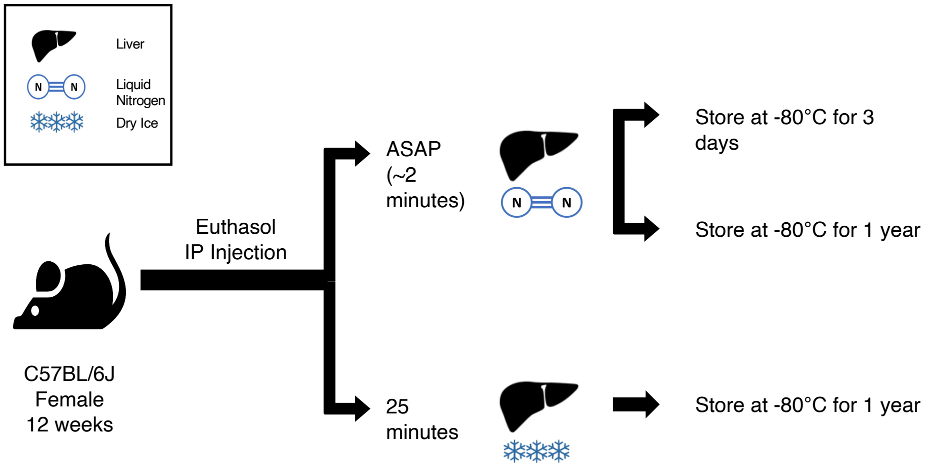

Figure S2. GLDS-49 Experimental Design. Related to Figure 1. Diagram of the ground-based preservation study comparing livers collected using standard laboratory protocols with livers collected from simulated spaceflight dissection flow and storage methods. Liver samples from twelve-week old female C57BL/6J mice were either snap frozen (n=3), snap frozen after a 25 min delay and stored for 3 days (n=3), or snap frozen after a 25 min delay and stored for 1 year (n=3). RNA-seq data were then generated using a polyA enrichment protocol.

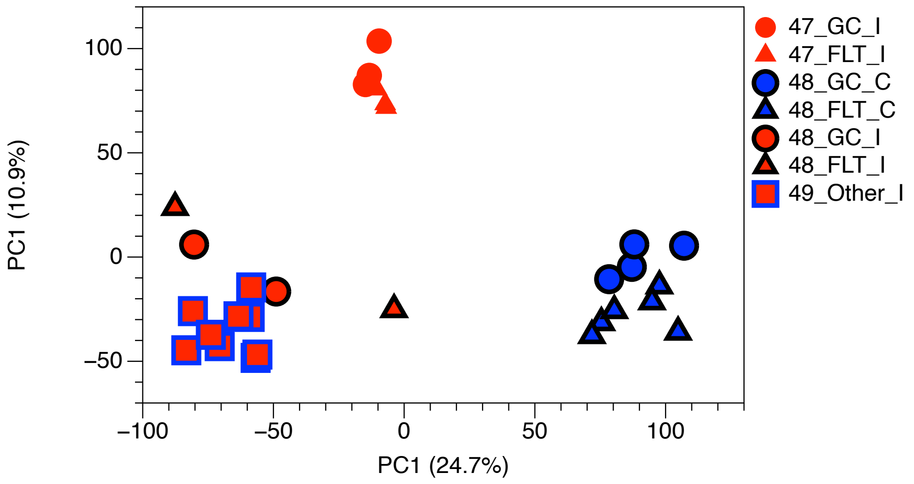

Outline:
None = GLDS-47
Black = GLDS-48
Blue = GLDS-49

Fill:
Red = Immediate (I)
Blue = Carcass (C)

Shape:
Triangle = Flight (FLT)
Circle = Ground control (GC)
Square = Other ground study (Other)

Figure S3. Carcass liver samples cluster together and away from immediate liver samples across datasets. Related to Figure 1. Principal component analysis containing data from RR-1 NASA spaceflight (FLT) and respective ground control (GC) carcass (48_FLT_C and 48_GC_C) and immediate (48_FLT_I and 48_GC_I) samples (GLDS-48), RR-1 CASIS FLT (47_FLT_I) and GC (47_GC_I) immediate samples (GLDS-47), and samples from a ground-based study (GLDS-49) in which livers were dissected immediately after euthanasia then frozen on either dry ice or submerged in liquid nitrogen then stored at -80 °C for 3 days or 1 year prior to processing (49_Other_I). Fill colors represent carcass (blue) or immediate (red) samples and the outline colors represent the GLDS dataset each sample came from (no outline = GLDS-47, black outline = GLDS-48, and blue outline = GLDS-49). Data are from GLDS-47, -48, and -49.

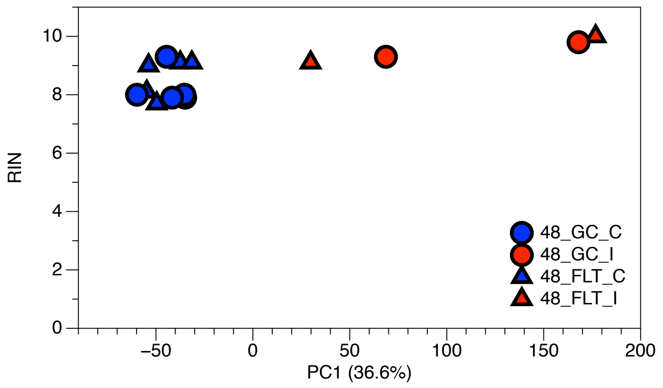

Figure S4. RNA Integrity Number analysis of Carcass and Immediate Liver samples. Related to Figures 1&3. RNA Integrity Numbers (RIN) for spaceflight (FLT) and ground control (GC) immediate (I) and carcass (C) samples plotted against principal component 1 (PC1) calculated from gene expression data. Data are from GLDS-48.

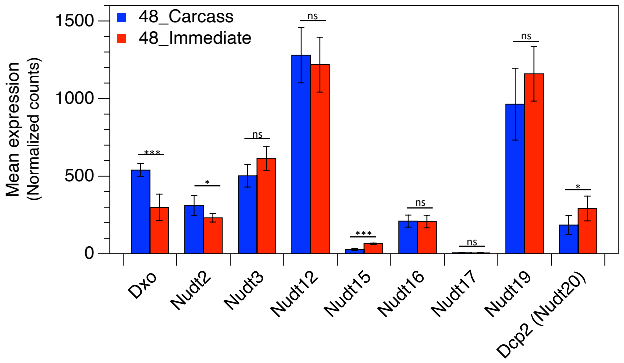

Figure S5. Expression of decapping enzymes. Related to Figure 3. Average (mean) expression of decapping enzymes in Carcass (blue) and Immediate (red) samples. Error bars indicate standard deviation (* = adj. $p < 0.05$, ** = adj. $p < 0.01$, *** = adj. $p < 0.001$, ns = not significant, Wald test). Data are from GLDS-48.

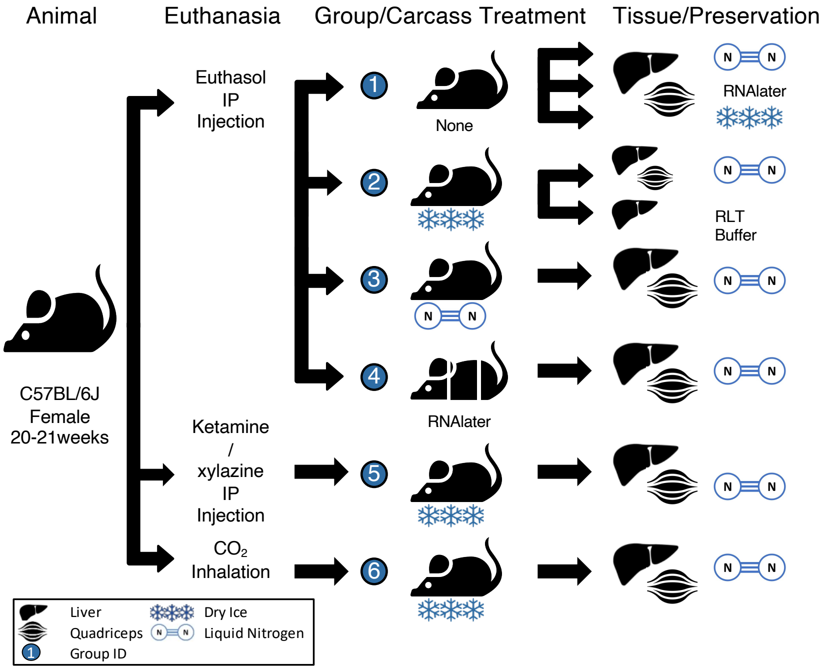

Figure S6. Ground-based tissue preservation study workflow. Related to Figure 5 and Tables 2&3. Diagram of the ground-based tissue preservation study to evaluate differences in indicated euthanasia and carcass and tissue preservation methods. Mice were euthanized with either pentobarbital/phenytoin (Euthasol®) or ketamine/xylazine injection, or CO_2 inhalation. Intact carcasses were preserved by freezing in liquid nitrogen or on dry ice, or by segmentation (head, chest, abdomen) and immersion in an ammonium sulfate solution (RNA*later*™). Carcasses were then thawed, and livers and quadriceps dissected and preserved in liquid nitrogen or guanidinium thiocyanate solution (Qiagen® RLT buffer). Alternatively, livers and quadriceps were dissected immediately and preserved by freezing in liquid nitrogen or on dry ice, or by immersion in an ammonium sulfate solution (RNA*later*™).

A

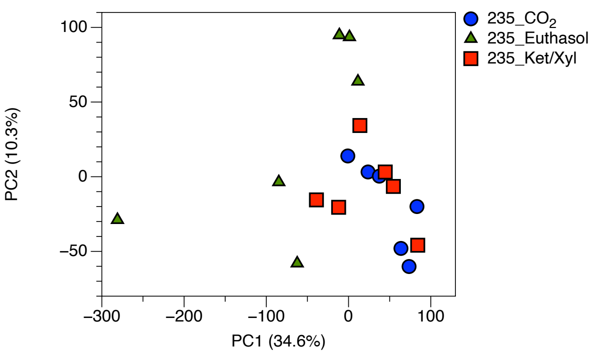

B

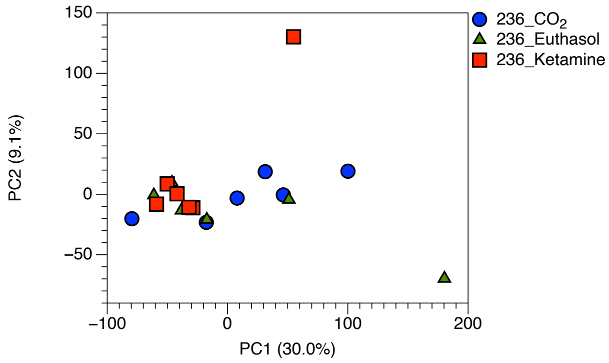

C

Comparison of euthanasia methods for Carcass liver samples (GLDS-235)					
Comparison	# DEG (adj. p < 0.05)	# DEG (adj. p < 0.05 & ILog2 FCI > 1.5)	# Enriched GO terms (NOM p < 0.01)	# Enriched GO terms (FDR < 0.5 & NOM p < 0.01)	# Enriched GO terms (FDR < 0.25 & NOM p < 0.01)
CO2_C_DI (n=6) v Euth_C_DI (n=6)	192	168	0, 42	0, 0	0, 0
CO2_C_DI (n=6) v Ket-Xyl_C_DI (n=6)	0	0	0, 51	0, 0	0, 0
Euth_C_DI (n=6) v Ket-Xyl_C_DI (n=6)	67	49	37, 0	1, 0	0, 0

D

Comparison of euthanasia methods for Carcass quadriceps samples (GLDS-236)					
Comparison	# DEG (adj. p < 0.05)	# DEG (adj. p < 0.05 & ILog2 FCI > 1.5)	# Enriched GO terms (NOM p < 0.01)	# Enriched GO terms (FDR < 0.5 & NOM p < 0.01)	# Enriched GO terms (FDR < 0.25 & NOM p < 0.01)
CO2_C_DI (n=6) v Euth_C_DI (n=6)	2	2	8, 23	2, 0	1, 0
CO2_C_DI (n=6) v Ket-Xyl_C_DI (n=6)	0	0	5, 100	2, 99	0, 4
Euth_C_DI (n=6) v Ket-Xyl_C_DI (n=6)	3	3	3, 64	0, 10	0, 0

E

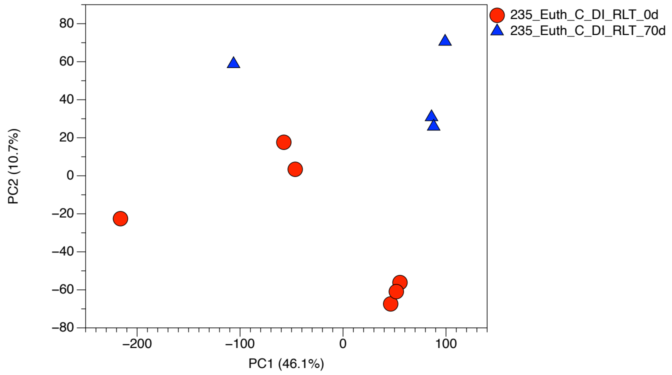

F

Tissue storage methods vs. RNAlat and LN2 preserved Immediate liver samples (GLDS-235)					
Comparison	# DEG (adj. p < 0.05)	# DEG (adj. p < 0.05 & ILog2 FCI > 1.5)	# Enriched GO terms (NOM p < 0.01)	# Enriched GO terms (FDR < 0.5 & NOM p < 0.01)	# Enriched GO terms (FDR < 0.25 & NOM p < 0.01)
Euth_C_DI_RLT-70d (n=4) v Euth_C_DI_RLT-0d (n=6)	284	174	3, 110	0, 31	0, 2
Euth_C_DI_RLT-70d (n=4) v Euth_I_LN2 (n=6)	1523	1250	67, 14	0, 0	0, 0
Euth_C_DI_RLT-70d (n=4) v Euth_I_RL (n=6)	1003	752	62, 18	3, 0	0, 0
Euth_C_DI_RLT-0d (n=6) v Euth_I_LN2 (n=6)	3798	3143	16, 0	0, 0	0, 0
Euth_C_DI_RLT-0d (n=6) v Euth_I_RL (n=6)	930	718	40, 1	1, 0	1, 0

Figure S7. Comparison of gene expression and gene ontology in liver and quadriceps samples derived from mice euthanized with different methods. Related to Figure 5 and Tables 2&3. Liver and quadriceps samples dissected from partially thawed frozen carcasses of mice that were euthanized with pentobarbital/phenytoin (Euthasol®), ketamine/xylazine, or carbon dioxide inhalation were evaluated for global gene expression differences via principal component analysis (A, liver and B, quadriceps), and the number of differentially expressed genes (DEG) and enriched gene ontology (GO) terms identified by Gene Set Enrichment Analysis via pairwise comparisons (phenotype permutation) (C, liver and D, quadriceps). Liver samples dissected from partially thawed frozen carcasses of mice that were euthanized with pentobarbital/phenytoin (Euthasol®), then snap frozen in liquid nitrogen and stored at -80 °C or homogenized in RLT buffer then stored for 70 d at -80 °C were evaluated for E) global gene expression differences via principal component analysis, and F) the number of differentially expressed genes (DEG) and enriched gene ontology (GO) terms identified by Gene Set Enrichment Analysis (phenotype permutation) via pairwise comparisons with immediate samples preserved in liquid nitrogen or RNAlater. For GO terms, the number on the left corresponds to the group to the left of the 'vs.', and number on the right corresponds to the group to the right of the 'vs.' in the "Comparison" column. n numbers, p values, log2 fold changes, and FDR values are indicated. Euth=euthanasia by pentobarbital/phenytoin (Euthasol®), Ket-Xyl= euthanasia by ketamine/xylazine, CO2=euthanasia by carbon dioxide inhalation, C=tissue dissected from frozen carcass that has been partially thawed, I=tissue dissected immediately after euthanasia, DI=dry ice, LN2=liquid nitrogen, RL=RNA*later*™. Liver and quadriceps data are from GLDS-235 and GLDS-236, respectively.

Supplementary Tables

Table S1. Liver samples analyzed in the ground-based tissue preservation study (GLDS-235). Related to Figure 5A&B and Table 2.

Group	Euthanasia	Carcass preservation	Tissue preservation	Mouse ID
**1**	Pentobarbital/phenytoin	None	Liquid nitrogen n=6<br>RNA*later*™ n=6<br>Dry ice n=6	M1-M6
**2**	Pentobarbital/phenytoin	Dry ice	Liquid nitrogen n=6<br>RLT buffer 70d at -80 °C	M7-M12
**3**	Pentobarbital/phenytoin	Liquid nitrogen	Liquid nitrogen n=5	M13-M18
**4**	Pentobarbital/phenytoin	RNA*later*™[a]	Liquid nitrogen n=5	M19-M24
**5**	Ketamine/xylazine	Dry ice	Liquid nitrogen n=6	M25-M30
**6**	Carbon dioxide	Dry ice	Liquid nitrogen n=6	M31-M36

a. Post carcass segmentation

Table S2. Quadriceps samples analyzed in the ground-based tissue preservation study (GLDS-236). Related to Figure 5C&D and Table 3.

Group	Euthanasia	Carcass preservation	Tissue preservation	Mouse ID
1	Pentobarbital/phenytoin	None	Liquid nitrogen n=6	M1-M6
			RNA*later*™ n=6	
			Dry ice n=6	
2	Pentobarbital/phenytoin	Dry ice	Liquid nitrogen n=6	M7-M12
3	Pentobarbital/phenytoin	Liquid nitrogen	Liquid nitrogen n=6	M13-M18
4	Pentobarbital/phenytoin	RNA*later*™[a]	Liquid nitrogen n=6	M19-M24
5	Ketamine/xylazine	Dry ice	Liquid nitrogen n=6	M25-M30
6	Carbon dioxide	Dry ice	Liquid nitrogen n=6	M31-M36

a. Post carcass segmentation

Table S3. Comparisons of immediate preservation methods on gene expression in livers. Related to Figure 5A&B and Table 2. The number of differentially expressed genes (DEG) and enriched gene ontology (GO) terms identified by Gene Set Enrichment Analysis (phenotype permutation) were evaluated pairwise in liver samples from different immediate preservation methods. For GO terms, the first number corresponds to the group to the left of the 'vs.', and second number corresponds to the group to the right of the 'vs.' in the "Comparison" column. n numbers, p values, log2 fold changes, and FDR values are indicated. Euth=euthanasia by pentobarbital/phenytoin, I=tissue dissected immediately after euthanasia, DI=dry ice, LN2=liquid nitrogen, RL=RNA*later*™. Data are from GLDS-235.

Comparison	# DEG (adj. p < 0.05)	# DEG (adj. p < 0.05 & |Log2 FC| > 1.5)	# Enriched GO terms (NOM p < 0.01)	# Enriched GO terms (FDR < 0.5 & NOM p < 0.01)	# Enriched GO terms (FDR < 0.25 & NOM p < 0.01)
**Euth_I_DI (n=6) vs. Euth_I_LN2 (n=6)**	16	16	31, 6	7, 0	0, 0
**Euth_I_DI (n=6) vs. Euth_I_RL (n=6)**	0	0	14, 8	0, 0	0, 0
**Euth_I_LN2 (n=6) vs. Euth_I_RL (n=6)**	14	14	3, 15	0, 0	0, 0

Table S4. Comparisons of carcass preservation methods to immediate RNA*later*™ method on gene expression in livers. Related to Figure 5A&B and Table 2. The number of differentially expressed genes (DEG) and enriched gene ontology (GO) terms identified by Gene Set Enrichment Analysis (phenotype permutation) were evaluated pairwise in liver samples from different carcass preservation methods compared with immediate samples preserved in RNA*later*™. For GO terms, the first number corresponds to the group to the left of the 'vs.', and the second number corresponds to the group to the right of the 'vs.' in the "Comparison" column. n numbers, p values, log2 fold changes, and FDR values are indicated. Euth=euthanasia by pentobarbital/phenytoin, I=tissue dissected immediately after euthanasia, C=tissue dissected from frozen carcass that has been partially thawed, DI=dry ice, LN2=liquid nitrogen, RL= RNA*later*™. Data are from GLDS-235.

**Comparison**	# DEG (adj. p < 0.05)	# DEG (adj. p < 0.05 & |Log2 FC| > 1.5)	# Enriched GO terms (NOM p < 0.01)	# Enriched GO terms (FDR < 0.5 & NOM p < 0.01)	# Enriched GO terms (FDR < 0.25 & NOM p < 0.01)
**Euth_C_DI (n=6) vs. Euth_I_RL (n=6)**	930	718	40, 1	1, 0	1, 0
**Euth_C_LN2 (n=5) vs. Euth_I_RL (n=6)**	197	118	90, 0	2, 0	0, 0
**Euth_C_RL (n=5) vs. Euth_I_RL (n=6)**	131	123	65, 0	0, 0	0, 0

Table S5. Comparisons of immediate preservation methods on gene expression in quadriceps. Related to Figure 5C&D and Table 3. The number of differentially expressed genes (DEG) and enriched gene ontology (GO) terms identified by Gene Set Enrichment Analysis (phenotype permutation) were evaluated pairwise in quadriceps samples from different immediate preservation methods. For GO terms, number on the left corresponds to the group to the left of the 'vs.', and number on the right corresponds to the group to the right of the 'vs.' in the "Comparison" column. n numbers, p values, log2 fold changes, and FDR values are indicated. Euth=euthanasia by pentobarbital/phenytoin, I=tissue dissected immediately after euthanasia, DI=dry ice, LN2=liquid nitrogen, RL= RNA*later*™. Data are from GLDS-236.

**Comparison**	# DEG (adj. p < 0.05)	# DEG (adj. p < 0.05 & |Log2 FC| > 1.5)	# Enriched GO terms (NOM p < 0.01)	# Enriched GO terms (FDR < 0.5 & NOM p < 0.01)	# Enriched GO terms (FDR < 0.25 & NOM p < 0.01)
**Euth_I_DI (n=6) vs. Euth_I_LN2 (n=6)**	2	1	2, 51	1, 2	0, 2
**Euth_I_DI (n=6) vs. Euth_I_RL (n=6)**	14	9	25, 26	6, 0	2, 0
**Euth_I_LN2 (n=6) vs. Euth_I_RL (n=6)**	0	0	66, 3	51, 0	0, 0

Table S6. Comparisons of carcass preservation methods to immediate RNA*later*™ method on gene expression in quadriceps. Related to Figure 5C&D and Table 3. The number of differentially expressed genes (DEG) and enriched gene ontology (GO) terms identified by Gene Set Enrichment Analysis (phenotype permutation) were evaluated pairwise in quadriceps samples from different carcass preservation methods compared with immediate samples preserved in RNA*later*™. For GO terms, number on the left corresponds to the group to the left of the 'vs.', and number on the right corresponds to the group to the right of the 'vs.' in the "Comparison" column. n numbers, p values, log2 fold changes, and FDR values are indicated. Euth=euthanasia by pentobarbital/phenytoin, I=tissue dissected immediately after euthanasia, C=tissue dissected from frozen carcass that has been partially thawed, DI=dry ice, LN2=liquid nitrogen, RL= RNA*later*™. Data are from GLDS-236.

**Comparison**	# DEG (adj. p < 0.05)	# DEG (adj. p < 0.05 & lLog2 FCl > 1.5)	# Enriched GO terms (NOM p < 0.01)	# Enriched GO terms (FDR < 0.5 & NOM p < 0.01)	# Enriched GO terms (FDR < 0.25 & NOM p < 0.01)
**Euth_C_DI (n=6) vs. Euth_I_RL (n=6)**	50	27	80, 5	67, 1	33 (41 with p < 0.05), 0
**Euth_C_LN2 (n=6) vs. Euth_I_RL (n=6)**	282	139	54, 8	4, 0	0, 0
**Euth_C_RL (n=6) vs. Euth_I_RL (n=6)**	59	40	135, 3	135, 1	34 (41 with p < 0.05), 0

Transparent Methods

Rodent Research-1 (RR-1) Study

Animals

As described previously (Choi et al., 2020), the study followed recommendations in the Guide for the Care and Use of Laboratory Animals (National Research Council, 2011) and was approved on May 21, 2014 by the Institutional Animal Care and Use Committee (IACUC) at NASA Ames Research Center and Kennedy Space Center (Protocol number NAS-13-002-Y1).

Spaceflight Mission

Rodent Research-1 (RR-1) was the first mission in which animals were maintained on the ISS for a long duration mission in the Rodent Habitat modified from heritage Animal Enclosure Module (AEM) hardware. Complete details were published previously (Choi et al., 2020). In short, RR-1 consisted of two experiments: ISS National Lab study (RR-1 CASIS) and NASA Validation study (RR-1 NASA). In the ISS National Lab Study, ten 32-week-old female C57BL/6NTac mice (Taconic Biosciences, Rensselaer, NY) were flown to space for 20-21 days then euthanized via IP injection of pentobarbital/phenytoin (Euthasol®) and dissected onboard the ISS. Livers were dissected then inserted into cryovials, which were then frozen in a cold stowage container that was pre-chilled to -130 °C before transferring to the Minus Eighty-Degree Laboratory Freezer (MELFI) at the end of each dissection session (-80 °C). In the NASA Validation study, ten 16-week-old female C57BL/6J mice (Jackson laboratories, Bar harbor, ME) were flown to the ISS for 37 days before euthanasia and subsequent dissection. Due to crew time constraint, only two (out of ten) mice were dissected immediately after euthanasia via IP injection of pentobarbital/phenytoin (Euthasol®) to recover spleen and liver tissues on the ISS. Isolated livers were preserved by using the same method as the ISS National Lab study livers. The remaining eight animals were euthanized, then intact carcasses were wrapped in aluminum foil, put in Ziploc bags, placed in a pre-chilled cold stowage container and stored in the MELFI. For both the ISS National Lab study and the NASA Validation study, there were respective cohorts of age-matched basal animals which were euthanized one day after launch as a baseline control as well as age-matched ground control animals kept in an ISS Environmental Simulator at Kennedy Space Center (KSC) on a 4-day delay to mimic spaceflight conditions. In addition, the NASA Validation study also had a cohort of age-matched vivarium control animals that were housed in the vivarium cages and followed the same experimental timeline and process as the spaceflight animals. A timeline indicating major events in the RR-1 mission is shown in Figure S1.

Sample Collection

The frozen intact carcasses from the NASA Validation study were partially thawed then dissected at NASA Ames Research Center upon return to Earth. One lobe of liver from each carcass was removed, immediately homogenized in RLT buffer (Qiagen, Valencia, CA) followed by snap freezing the tissue homogenates in LN2. Quadriceps were snap frozen upon collection. Tissues were stored at -80 °C until extraction.

RNA Isolation

RNA was isolated from all liver and quadriceps samples using the following methods. For the liver samples, RNA was extracted with the AllPrep DNA/RNA Mini Kit (Qiagen, Valencia, CA) following the

manufacturer's protocol. Briefly, homogenization buffer was made by adding 1:100 volume ratio of beta-mercaptoethanol to RLT buffer and kept on ice until use. Approximately 30 mg of tissue was cut using a sterile scalpel and immediately placed in 800 µL of the RLT buffer solution. Each sample was then homogenized for approximately 20 seconds at 21,000 RPM using a Polytron PT1300D handheld homogenizer with a 5 mm standard dispersing aggregate tip (Kinematica, Bohemia, NY). Homogenates were centrifuged for 3 minutes at room temperature at 15,000 RPM to remove cell debris. The supernatant from each sample was used to isolate and purify RNA following the manufacturer's protocol including on-column DNase treatment with RNase-free DNase (Qiagen, Valencia, CA). RNA was eluted twice per sample in 30 µL RNase- and DNase-free H2O per elution. For quadriceps samples, RNA was extracted using TRIzol reagents (Thermo Fisher Scientific, Waltham, MA) according to the manufacturer's protocol, and the isolated RNA samples were then treated on column with RNase-free DNase (Qiagen, Valencia, CA) and RNeasy Mini kit (Qiagen, Valencia, CA). Concentration and absorbance ratios of all the isolated liver and quadriceps RNA samples were measured using the NanoDrop 2000 spectrophotometer (Thermo Fisher Scientific, Waltham, MA). RNA quality was assessed using the Agilent 2100 Bioanalyzer with the Agilent RNA 6000 Nano Kit or Agilent RNA 6000 Pico Kit (Agilent Technologies, Santa Clara, CA).

Library Preparation and RNA-Sequencing

Samples with RNA Integrity Number (RIN) of 6 or above were sent to the University of California (UC), Davis Genome Center where the libraries were constructed, and RNA-sequencing was performed. All the RR-1 RNA-sequencing data analyzed in this manuscript were obtained from the NASA GeneLab Data Repository (https://genelab.nasa.gov/), including GLDS-47, GLDS-48, and GLDS-168. The RR-1 liver RNA samples were sequenced twice. First, libraries were generated using the Illumina TruSeq Stranded RNA library prep kit (Illumina, San Diego, CA) after polyA selection, and sequencing was done with 50 bp single end reads on the Illumina HiSeq 3000 platform (GLDS-47 and GLDS-48). Second, selected RNA samples were spiked in with ERCC ExFold RNA Spike-In Mixes (Thermo Fisher Scientific, Waltham, MA) before shipping to the UC Davis Genome Center. Ribosomal RNA was removed with the Illumina RiboZero Gold ribodepletion kit then RNA sequencing libraries were constructed using the KAPA RNA HyperPrep kit (Roche, Basel, Switzerland) and the sequencing was done with 150 bp paired end reads on the Illumina HiSeq 4000 platform (GLDS-168).

Ground-based Tissue Preservation Study

Animals

20- to 21-week-old female C57BL/6J mice (Jackson laboratories, Bar harbor, ME) were shipped to the NASA Ames Research Center Animal Care Facility and were randomly housed in the standard vivarium cages with up to five mice per cage. The animals were acclimated for five days before the start of procedures to ensure recovery from the transportation stress. During acclimation, the animals were maintained on a 12h light/dark cycle and were provided with standard chow and water access *ad libitum*. One day before euthanasia, animal body weights were measured and used to distribute the animals into six groups (n=6/group) with similar average body weights. Animal health status, water and food intake were monitored daily. The study followed recommendations in the Guide for the Care and Use of Laboratory Animals (National Research Council, 2011) and was approved on February 8, 2018 by the Institutional Animal Care and Use Committee (IACUC) at NASA Ames Research Center (Protocol number NAS-17-006-Y1).

Animal Euthanasia and Dissection

The detailed descriptions and rationale of each group are as follows as well as outlined in Figure S6 and Tables S1 and S2. Group 1 (M1-M6) animals were euthanized by intraperitoneal injection of pentobarbital/phenytoin (Euthasol®) (80 mg in 0.2 ml) (Virbac, West Lake, TX) followed by cervical dislocation. Dissection was performed immediately after euthanasia without freezing the carcasses. Left lobes of livers and quadriceps were subdivided into three sections and each tissue section was preserved either by freezing on dry ice, snap freezing in liquid nitrogen, or preserved in RNA*later*™ solution (Thermo Fisher Scientific, Waltham, WA). For the tissue sections preserved in RNA*later*™, tissue sections were submerged in RNA*later*™ at 4 °C for 3 days then frozen and stored at -80 °C. Note that this is the only group of animals that were dissected upon euthanasia. Carcasses from animals in subsequent groups were preserved intact using various methods then dissected at a later date. Group 1 tissue sections that were preserved by freezing on dry ice most closely mimics the process that was used to generate RR-1 NASA and CASIS immediate samples.

Group 2 (M7-M12) animals were euthanized by intraperitoneal injection of pentobarbital/phenytoin (Euthasol®) followed by cervical dislocation. The carcasses were wrapped in foil and preserved by freezing on dry ice, similar to the intact carcass preservation method used for the RR-1 NASA Validation Study. Once frozen, the carcasses were stored at -80 °C. On the day of dissection, mouse carcasses were removed from the -80 °C freezer and thawed at room temperature for 15 to 20 minutes prior to dissection. Left lobes of livers were removed and divided into two: one piece was snap frozen in liquid nitrogen then stored at -80 °C; the other piece was homogenized in RLT buffer (Qiagen, Valencia, CA) and the tissue homogenate was snap frozen in liquid nitrogen then stored at -80 °C for 70 days before RNA extraction to simulate the process used to generate the RR-1 NASA "carcass" liver samples. This extended storage did not result in a substantial number of DEG (Figure S7E&F). Quadriceps were snap frozen in liquid nitrogen after dissection then stored at -80 °C.

Group 3 (M13-M18) animals were euthanized by intraperitoneal injection of pentobarbital/phenytoin (Euthasol®) followed by cervical dislocation. The intact carcasses were wrapped in foil and preserved by snap freezing in liquid nitrogen followed by storage at -80 °C. On the day of dissection, mouse carcasses were removed from the -80 °C freezer and thawed at room temperature for 15 to 20 minutes prior to dissection. Left lobes of livers and quadriceps were collected and snap frozen in liquid nitrogen then stored at -80 °C.

Group 4 (M19-M24) animals were euthanized by intraperitoneal injection of pentobarbital/phenytoin (Euthasol®) followed by cervical dislocation. The carcasses were sectioned into 3 sections, head, chest, and abdomen with tail removed and discarded and each part was submerged in RNA*later*™ solution (Thermo Fisher Scientific, Waltham, WA) and placed at 4 °C for 3 days to allow thorough permeation before being stored at -80 °C. On the day of dissection, mouse carcasses were removed from the -80 °C freezer and thawed at room temperature for 15 to 20 minutes prior to dissection. Left lobes of livers and quadriceps were collected and snap frozen in liquid nitrogen then stored at -80 °C. This group was used to simulate the procedure done in the RR-7 mission and to test if gene expression signals could be better preserved using an RNA-specific preservative.

Group 5 (M25-30) animals were euthanized by intraperitoneal injection of ketamine/xylazine (10mg/mL / 3mg/mL in 0.3mL PBS) followed by cervical dislocation. The intact carcasses were wrapped in foil and preserved by freezing on dry ice then stored at -80 °C. On the day of dissection, mouse carcasses were removed from the -80 °C freezer and thawed at room temperature for 15 to 20 minutes prior to

dissection. Left lobes of livers and quadriceps were collected and snap frozen in liquid nitrogen then stored at -80 °C. The euthanasia and preservation methods used in this group mimics the process used to generate RR-3 carcass liver samples (Smith et al., 2017). Ketamine/xylazine is currently the most common euthanasia method used in RR missions.

Group 6 (M31-M36) animals were euthanized by carbon dioxide inhalation followed by cervical dislocation. The carcasses were wrapped in foil and preserved by freezing on dry ice then stored at -80 °C. On the day of dissection, mouse carcasses were removed from the -80 °C freezer and thawed at room temperature for 15 to 20 minutes prior to dissection. Left lobes of livers and quadriceps were collected and snap frozen in liquid nitrogen then stored at -80 °C. This group represents the euthanasia method commonly used in terrestrial laboratories and was used to evaluate any effects on gene expression due to the drug-induced euthanasia methods that have been used in RR missions.

RNA Isolation

RNA was isolated from partial left liver lobe and partial quadriceps muscle tissues using the AllPrep DNA/RNA Mini Kit (Qiagen, Valencia, CA). Briefly, homogenization buffer was made by adding 1:100 volume ratio of beta-mercaptoethanol to RLT buffer and kept on ice until use. On average, 48.85 mg of left liver lobe and 20.08 mg of left or right quadriceps was cut using a sterile scalpel and immediately placed in 600 μL of the RLT buffer solution. Complete tissue dispersion was achieved using the hand-held Polytron PT1300D homogenizer with 5 mm standard dispersing aggregate by implementing 20 second homogenization periods at a speed of 20,000 RPM. Homogenized samples were centrifuged for 3 minutes at room temperature at 15,000 RPM to remove cell debris. The supernatant from each sample was used to isolate and purify RNA following the manufacturer's protocol. RNA was treated with RNase-Free DNase (Qiagen, Valencia, CA) and eluted in 50 μL of RNase- and DNase-free H_2O molecular grade water. RNA concentration was measured using Qubit 3.0 Fluorimeter (Thermo Fisher Scientific, Waltham, MA). RNA quality was assessed using the Agilent 2100 Bioanalyzer with the Agilent RNA 6000 Nano Kit or Agilent RNA 6000 Pico Kit (Agilent Technologies, Santa Clara, CA).

Library Preparation and Sequencing

Three microliters of Mix 1 or Mix 2 of ERCC ExFold RNA Spike-In (Thermo Fisher Scientific, Waltham, MA) at a dilution of 1:100 was added to 1.5 μg aliquots of RNA immediately after extraction. The two mixes were randomly distributed within the six experimental groups. In addition, Universal Human and Mouse Reference RNA samples (Agilent Technologies, Santa Clara, CA) were included as control samples in the library construction and sequencing.

Library construction was performed using 500 ng of ERCC-spiked total RNA with an average RIN of 7.8 for liver samples and 9.8 for quadriceps samples. Total RNA was depleted of the ribosomal fraction and libraries were constructed with TruSeq Stranded Total RNA with Ribo-Zero Gold kit (Illumina, San Diego, CA). Libraries were indexed using 1.5 μM Unique Dual Index adapters with Unique Molecular Identifiers (Integrated DNA Technologies, Coralville, IA) and 15 cycles of amplification were performed to reach desired library concentration. Library size was assessed with 4200 TapeStation (Agilent Technologies, Santa Clara, CA), targeting average size of 300 nt.

Libraries were multiplexed then quantified using Universal qPCR Master Mix (Kapa Biosystems, Wilmington, MA). The library pool was sequenced on an iSeq 100 (Illumina, San Diego, CA) to assess sample quality and pool balancing before large-scale sequencing. The final library pool (with 1% PhiX

spike-in for instrument control) was sequenced on a NovaSeq 6000 using one S4 and one S2 Reagent Kit (Illumina, San Diego, CA), paired-end and 149 bp reads, targeting 60 million clusters for each experimental sample.

GLDS-49 Ground-based Freezing Study

Animals

As described previously (Choi et al., 2020), the study followed recommendations in the Guide for the Care and Use of Laboratory Animals (National Research Council, 2011) and was approved on May 21, 2014 by the Institutional Animal Care and Use Committee (IACUC) at NASA Ames Research Center and Kennedy Space Center (Protocol number NAS-13-002-Y1).

To compare standard laboratory protocols for tissue freezing and storage with a spaceflight timeline-simulated liver dissection and long-term storage, liver samples from twelve-week old female C57BL/6J mice (Jackson laboratories, Bar harbor, ME) were either immediately snap frozen (in liquid nitrogen), snap frozen after a 25 min delay and stored for 3 days, or snap frozen after a 25 min delay and stored for 1 year (Figure S2).

Sample Collection

The liver tissues of twelve-week-old C57BL/6J mice (Jackson laboratories, Bar harbor, ME) were received from the Rodent Research project collected in a ground-based preservation and storage study (Choi et al., 2016). Three groups of livers were included: 1) Liver tissues dissected and frozen on dry ice 25 min after euthanizing with pentobarbital/phenytoin (Euthasol®) followed by cervical dislocation. At the time of RNA extraction, the liver tissues had been stored at -80 °C for around 1-year; 2) Liver tissues dissected 3 min after euthanizing with pentobarbital/phenytoin (Euthasol®) followed by cervical dislocation and snap-freezing in liquid nitrogen. At the time of RNA extraction, the liver tissues had been stored at -80 °C for around 1-year; 3) Liver tissues dissected 3 minutes after euthanizing with pentobarbital/phenytoin (Euthasol®) followed by cervical dislocation and snap-freezing in liquid nitrogen. At the time of RNA extraction, the liver tissues had been stored at -80 °C for only 3 days. This group served as a positive control for delayed dissection and long-term storage.

RNA Isolation

RNA was isolated using the AllPrep DNA/RNA Mini Kit (Qiagen, Valencia, CA) following the manufacturer’s protocol. Briefly, homogenization buffer was made by adding 1:100 volume ratio of beta-mercaptoethanol to RLT buffer and kept on ice until use. Approximately 30 mg of tissue was cut using a sterile scalpel and immediately placed in 800 µL of the RLT buffer solution. Each sample was then homogenized for approximately 20 seconds at 21,000 RPM using a Polytron PT1300D handheld homogenizer with a 5 mm standard dispersing aggregate tip (Kinematica, Bohemia, NY). Homogenates were centrifuged for 3 minutes at room temperature at 15,000 RPM to remove cell debris. The supernatant from each sample was used to isolate and purify RNA following the manufacturer's protocol including on-column DNase treatment with RNase-free DNase (Qiagen, Valencia, CA). RNA was eluted twice per sample in 30 µL RNase- and DNase-free H_2O per elution. Concentration and absorbance ratios were measured using the NanoDrop 2000 spectrophotometer (Thermo Fisher Scientific, Waltham, MA).

RNA quality was assessed using the Agilent 2100 Bioanalyzer with the Agilent RNA 6000 Nano Kit (Agilent Technologies, Santa Clara, CA).

Library Preparation and RNA-Sequencing

Samples with RNA Integrity Number (RIN) of 9 or above were sent to the University of California (UC), Davis Genome Center where the libraries were constructed, and RNA-sequencing was performed. Libraries were generated using the Illumina TruSeq Stranded RNA library prep kit (Illumina, San Diego, CA) after polyA selection, and sequencing was done with 50 bp single end reads on the Illumina HiSeq 3000 platform.

RNA Sequencing Data Analysis

Raw RNA sequence data from the RR-1 NASA Validation flight liver (GLDS-48 and GLDS-168) samples, RR-1 CASIS liver samples (GLDS-47), and the ground-based studies designed to simulate and assess spaceflight euthanasia, carcass and tissue preservation, and/or storage protocols, GLDS-49, GLDS-235, and GLDS-236 were analyzed using the GeneLab standard RNAseq analysis pipeline (https://github.com/nasa/GeneLab_Data_Processing/tree/master/RNAseq). First, adapters were removed with Cutadapt (v2.3) (Martin, 2011) using the Trim Galore! (v0.6.2) wrapper. Raw and trimmed read quality were evaluated with FastQC (v0.11.8), and MultiQC (v1.7) was used to generate MultiQC reports. *Mus musculus* STAR and RSEM references were built using STAR (v2.7.1a) and RSEM (v1.3.1) (Li and Dewey, 2011), respectively, with ensembl genome version mm10-GRCm38 (Mus_musculus.GRCm38.dna.toplevel.fa), and the following gtf annotation file: Mus_musculus.GRCm38.96.gtf. Trimmed reads were aligned to the *Mus musculus* STAR reference with STAR (v2.7.1a) (Dobin et al., 2013) and aligned reads were quantified using RSEM (v1.3.1) (Li and Dewey, 2011).

The following samples were used for downstream analyses (abbreviations are consistent with the respective sample names in the GLDS Data Repository); GLDS-47: FLT and GC; GLDS-48: FLT_C, FLT_I, GC_C, and GC_I; GLDS-49: LN2_3d, LN2_1y, DI_1y; GLDS-168: RR1_FLT_wERCC and RR1_GC_wERCC; for GLDS-235 and GLDS-236, all samples indicated in Figure S6 and Tables S1 and S2 were included. For each GLDS dataset, quantification data from select samples were imported to R (v3.6.0) with tximport (v1.14.0) (Soneson et al., 2016) and normalized with DESeq2 (v1.26.0) (Love et al., 2014). All ERCC genes were removed prior to normalization. Differential expression analysis was performed in R (v3.6.0) using DESeq2 (v1.26.0) (Love et al., 2014); all groups were compared using the Wald test and the likelihood ratio test was used to generate the F statistic p-value. Gene annotations were assigned using the following Bioconductor and annotation packages: STRINGdb (Szklarczyk et al., 2019), PANTHER.db (Muller, 2017), and org.Mm.eg.db (Carlson, 2017).

Transcript Integrity Analysis

The geneBody_coverage.py function from RSeQC (v3.0.1) (Wang et al., 2012) was used to assess coverage across the median 1000 expressed genes across all datasets. A transcript integrity metric was defined as the ratio between the coverage in a window corresponding to position 10-30% and 80-100%

(relative to the entire gene length) and used in boxplots. To determine significance between groups the nonparametric Mann–Whitney U test was used as distributions were not normal.

Gene Set Enrichment Analysis

Pairwise gene set enrichment analysis (GSEA) was performed on the normalized counts from select samples in GLDS-48, GLDS-168, GLDS-235, and GLDS-236 using the C5: Gene Ontology (GO) gene set (MSigDB v7.1) as described (Subramanian et al., 2005). All comparisons were performed using the phenotype permutation, except those involving GLDS-48 immediate samples, which used the gene set permutation due to low sample size. The ranked lists of genes were defined by the signal-to-noise metric, and the statistical significance were determined by 1000 permutations of the gene set. FDR ≤ 0.25 and FDR ≤ 0.05 were considered significant for comparisons using the phenotype and gene set permutations, respectively, according to the authors' recommendation.

Supplemental References

Carlson, M. (2017). org.Mm.eg.db. Bioconductor. https://doi.org/10.18129/B9.BIOC.ORG.MM.EG.DB

Dobin, A., Davis, C.A., Schlesinger, F., Drenkow, J., Zaleski, C., Jha, S., Batut, P., Chaisson, M., Gingeras, T.R. (2013). STAR: ultrafast universal RNA-seq aligner. Bioinformatics 29, 15–21.

Li, B., Dewey, C.N. (2011). RSEM: accurate transcript quantification from RNA-Seq data with or without a reference genome. BMC Bioinformatics 12, 323.

Love, M.I., Huber, W., Anders, S. (2014). Moderated estimation of fold change and dispersion for RNA-seq data with DESeq2. Genome Biol 15, 550.

Martin, M. (2011). Cutadapt removes adapter sequences from high-throughput sequencing reads. EMBnet j. 17, 10.

Muller, J. (2017). PANTHER.db. Bioconductor. https://doi.org/10.18129/B9.BIOC.PANTHER.DB

National Research Council. (2011). Guide for the care and use of laboratory animals (National Academies Press).

Smith, R., Cramer, M., Globus, R., Galazka, J. (2017). Rodent Research-3-CASIS: Mouse liver transcriptomic, proteomic, and epigenomic data. GeneLab, Version 4, http://doi.org/10.26030/9k6w-4c28.

Soneson, C., Love, M.I., Robinson, M.D. (2016). Differential analyses for RNA-seq: transcript-level estimates improve gene-level inferences. F1000Res 4, 1521.

Subramanian, A., Tamayo, P., Mootha, V.K., Mukherjee, S., Ebert, B.L., Gillette, M.A., Paulovich, A., Pomeroy, S.L., Golub, T.R., Lander, E.S., Mesirov, J.P. (2005). Gene set enrichment analysis: A knowledge-based approach for interpreting genome-wide expression profiles. Proceedings of the National Academy of Sciences 102, 15545–15550.

Szklarczyk, D., Gable, A.L., Lyon, D., Junge, A., Wyder, S., Huerta-Cepas, J., Simonovic, M., Doncheva, N.T., Morris, J.H., Bork, P., Jensen, L.J., Mering, C. von. (2019). STRING v11: protein–protein association networks with increased coverage, supporting functional discovery in genome-wide experimental datasets. Nucleic Acids Research 47, D607–D613.

Wang, L., Wang, S., Li, W. (2012). RSeQC: quality control of RNA-seq experiments. Bioinformatics 28, 2184–2185.
